# Concussion of the Brain

**Published:** 1876-07

**Authors:** F. W. Mercer

**Affiliations:** Asst. Physician


### CONCUSSION OF THE BRAIN.

By F. W. MERCER, Asst. Physician.

Reported from No'es maje June, 1875, at Illinois Southern Hospital for the Insane.

J. C. W., æt. 14, an epileptic, while climbing a ladder from the barn floor to hay mow had a fit, and fell about ten feet, striking partly upon the vertex and right parietal region.

The attendant who saw him fall, went immediately to his assistance, and found him insensible. With little delay, the patient was carried to his ward and his condition reported. I saw him about twenty minutes after the injury; he was comatose; breathing, slow and somewhat labored; pulse slow, eyes closed, and pupils dilated. An examination disclosed nothing but free hæmorrhage from the right ear; he had already lost about three ounces of blood, and the flow from the ear continued steadily. Instruction given to keep him quiet in bed. Saw him again in the evening, about five hours after the injury; had been vomiting, and could be aroused with difficulty. Hæmorrhage from ear not so free, but accompanied by a large amount of watery fluid, saturating his pillow. Pulse, 100; temperature, 95°. Ordered cathartic and cold applications to head.

Morning of 3rd found him sitting up, and was informed by the attendant that the patient had insisted upon leaving his bed and had walked about the ward very unsteadily, catching at articles of furniture for support, and complaining that everything about him was spinning around. The bowels had moved during the night; the stomach had become quiet, and a large amount (several ounces) of bloody serum escaped from the ear; pulse, 120; temperature, 102°. Ordered continued rest in bed, cold to head, and carefully regulated nourishment. In the evening he appeared less restless, inclined to drowsiness, but easily aroused; pulse, 125; temperature, 103.2°; discharge from ear much diminished.

Morning of 4th. Patient said to have been restless first part of the night, but quiet and appeared to sleep after midnight; discharge from ear has ceased. His mind appears clear, and he answers questions well; wants to dress and be permitted to walk about the ward. Pulse, 98; temperature, 99°. Ordered continuation in bed, with cold to head.

Evening. He is quite talkative; no discharge from ear; pulse, 85; temperature, 99°.

Morning of 5th. He is out of bed; complains of dull headache; pulse, 90; temperature, 99°. Directed that he should be kept quiet; discontinued application to head.

Evening. pulse, 82; temperature, 99°.

6th. Out of bed this morning; pulse, 85°; temperature, normal.

8th. Is going about the house as usual; appears well.

